# Evidence-informed obstetric practice during normal birth in China: trends and influences in four hospitals

**DOI:** 10.1186/1472-6963-6-29

**Published:** 2006-03-08

**Authors:** Xu Qian, Helen Smith, Hong Liang, Ji Liang, Paul Garner

**Affiliations:** 1 Effective Health Care Research Programme, Department of Maternal and Child Health, Fudan University School of Public Health, 138 Yi Xue Yuan Road, Shanghai 200032, P.R. China; 2 Effective Health Care Research Programme, International Health Research Group, Liverpool School of Tropical Medicine, Liverpool, L3 5QA, UK

## Abstract

**Background:**

A variety of international organizations, professional groups and individuals are promoting evidence-informed obstetric care in China. We measured change in obstetric practice during vaginal delivery that could be attributed to the diffusion of evidence-based messages, and explored influences on practice change.

**Methods:**

Sample surveys of women at postnatal discharge in three government hospitals in Shanghai and one in neighbouring Jiangsu province carried out in 1999, repeated in 2003, and compared. Main outcome measures were changes in obstetric practice and influences on provider behaviour. "Routine practice" was defined as more than 65% of vaginal births. Semi-structured interviews with doctors explored influences on practice.

**Results:**

In 1999, episiotomy was routine at all four hospitals; pubic shaving, rectal examination (to monitor labour) and electronic fetal heart monitoring were routine at three hospitals; and enema on admission was common at one hospital. In 2003, episiotomy rates remained high at all hospitals, and actually significantly increased at one; pubic shaving was less common at one hospital; one hospital stopped rectal examination for monitoring labour, and the one hospital where enemas were common stopped this practice. Mobility during labour increased in three hospitals. Continuous support was variable between hospitals at baseline and showed no change with the 2003 survey.

Provider behaviour was mainly influenced by international best practice standards promoted by hospital directors, and national legislation about clinical practice.

**Conclusion:**

Obstetric practice became more evidence-informed in this selected group of hospitals in China. Change was not directly related to the promotion of evidence-based practice in the region. Hospital directors and national legislation seem to be particularly important influences on provider behaviour at the hospital level.

## Background

Evidence-based practice is widely promoted, but actual obstetric practice is often not evidence-informed. Research has shown large practice variations across facilities in the same country in China [1], South Africa [2] and the UK [3]; in particular, unnecessary obstetric procedures during normal birth are common and may actually be increasing in some countries [4-6]. Increased numbers of reliable summaries of scientific evidence globally has improved knowledge, but there remains a shortfall in uptake and use of this information [2]. Obstetricians continue to implement practices such as routine episiotomy that have been shown to be harmful, and fail to implement those with demonstrable benefit [1,2]. In addition, global Caesarean rates are increasing, probably as a result of women's preference [7-9].

Shanghai has the highest rate of institutional deliveries in China, and reached almost 100% in 2002 [10]. As hospital data are not routinely available, we measured indicators of evidence-informed obstetric practice in three hospitals in Shanghai and one in neighbouring Jiangsu province in 1999 using exit interviews: 70% or more of women experiencing normal birth in three of the four hospitals were subject to three practices that were not evidence-informed (pubic shaving, rectal examination, and episiotomy). Most women did not receive companionship during labour (shown by research to be effective); delivered lying down (other positions may be more effective) and none received pain relief [1]. For these measured indicators, then, practice was not generally research informed; in addition, the procedures cause the woman unnecessary discomfort and increase the cost of service provision [11].

The literature on changing clinician behaviour and practice is vast and many interventions, underpinned by social, organisational and management theories, have been evaluated [12,13]. Although individual interventions demonstrate some success when implemented on their own (for example using opinion leaders to promote use of evidence-informed guidelines) [14], change strategies that use more than one intervention appear to have a greater effect on practice [15]. Recent research suggests the uptake of research findings into routine practice is a chaotic and unpredictable process [16,17], and strategies that use dissemination and diffusion can promote evidence-informed practice via formal and informal mechanisms [18] that help localise decision-making [19].

The 1999 study was part of a range of activities around promotion of using systematic reviews to inform obstetric practice nationally in China and locally in Shanghai (table 1). Some were initiated by international agencies, some as part of the Effective Health Care Programme supported by DFID, and some by Professor Qian Xu as president of the women's health care division of the China Preventive Medicine Association. In the light of these policy and dissemination initiatives, we sought indicators of change in policy by examining change in clinical practice since the initial study in 1999, and explored potential influences on provider behaviour. In particular, we sought evidence that the multilateral initiatives or the local dissemination activities were perceived to influence practice.

## Methods

### Study sites

We repeated our sample surveys of practice at the same four hospitals as the 1999 study. In 1999, the hospitals had been purposefully selected to represent best practice and with national training institution responsibilities at different levels of service: a specialist (university-affiliated) hospital, a city Maternal and Child Health (MCH) hospital, an urban district hospital, and a rural county hospital. Average deliveries per month at these hospitals in 2003 was: 250 (specialist); 550 (MCH); 40 (district); and 150 (county). The baseline paper provides further details about the study sites [1]. As data on delivery procedures is not routinely available, we surveyed women using exit interviews after delivery.

There were small changes in the organisation of care at the hospitals during the study period (1999–2003); medical accident legislation for special examinations and operative procedures was introduced in 2002 [20], and the head of obstetrics changed at the county and specialist hospitals.

### Indicator practices

The study examined selected obstetric practices where there is reasonable evidence of benefit or harm from systematic reviews published on the WHO Reproductive Health Library. Systematic reviews show there is insufficient evidence to recommend routine pubic shaving [21], enemas [22] and episiotomy [23]; these practices cause discomfort, may be harmful and would not be routine in evidence-informed practice. Other practices such as continuous support for women during childbirth [24], non-supine position for delivery [25] and mobility during labour [26] demonstrate benefits to women and infants, and evidence-informed practice would promote their adoption. For the purpose of this study and based on local hospital policy, "continuous support during labour" is defined as presence of a husband, friend, family member or caregiver provided by the hospital; and "mobility" refers to women being able to walk freely whilst in the labour room. We documented practice rates using exit interviews with women, verified by checking patient notes, and asked explicitly about provider views of these procedures using semi structured interviews.

### Exit interviews

We conducted exit interviews at all sites using the same methods and questionnaire as the 1999 study [1]. 711 postpartum women with no serious obstetric complications were interviewed consecutively from November 2002 – July 2003; those who had experienced adverse birth outcomes (for example, perinatal death, abnormal baby) were not approached. Women were asked about use of the indicator practices, mode of delivery, and their views on the childbirth environment. Trained senior medical students visited the four hospitals each day over the study period to interview women due for discharge that day. Verbal consent was obtained from all participants; 5% of women refused to be interviewed, mainly because they did not want to be disturbed. As in the baseline survey, practice rates were determined by interviewing women and verified by hospital notes (for practices that are routinely recorded). The exit interview schedule was piloted with 10 women to ensure the questions were correctly understood, and note audit forms tested to ensure accurate recording. Hospital notes were complete at all study sites; procedures used in the delivery room are routinely recorded by staff on a structured form with tick boxes.

### Semi-structured interviews

Providers were interviewed about changes in policy, guidelines and service provider's function during the four years, their views about Caesarean section rates, interventions during childbirth (listed in table 4), constraints to good practice, and opportunities for changing practice. We purposefully selected 24 providers for interview; to ensure all levels of staff were included in the sample we interviewed three doctors (one senior, one junior and the doctor in charge) and three midwives (one senior, one junior and the midwife in charge) at each hospital. We arranged a convenient time for the interview with each provider, and none refused to participate. Researchers from MCH department of the School of Public Health, Fudan University, conducted all interviews, which lasted between 30–50 minutes and were tape-recorded. Verbal consent was obtained, and confidentiality was explained to all respondents.

### Data analysis

Data from exit interviews were double entered, checked and analysed using Epi Info [27], and we analysed a merged database of baseline and follow-up data using SPSS [28]. We used the Students t test to compare participants' age, and Chi^2 ^to determine any difference between participant characteristics and practice rates from 1999–2003. As women often did not know when the decision for Caesarean section was made, and as this decision influences most of the procedures being measured, we only report on indicator practices for women who had a vaginal birth (including vacuum and forceps delivery).

Data from semi-structured interviews were transcribed and analysed manually using methods of the Framework approach [29]. First LH compared written notes and the recorded version to make the data complete, and then listed all emergent questions, topics and concepts in a conceptual framework. LH and QX used this framework to code each transcript, then summarised the findings according to key categories and translated the summary into English. QX, HS, LH then discussed the summarised findings in light of their different disciplinary backgrounds (medicine, social science and public health), explored perspectives that deviated from the identified categories by referring back to the original transcripts for clarification, and came to a consensus on the main themes emerging from the dataset. We present typical quotes to illustrate participant views and perspectives.

### Ethical approval

The study was approved by Fudan University Institutional Review Board (IRB00002408, FWA00002399).

## Results

### Practice

We conducted 711 exit interviews with women across the four hospitals, and their characteristics are shown in table 2. Most were primigravidae (related to the one-child family planning policy); women at the rural county hospital tended to be younger, less educated, and uninsured. In comparison to the 1999 data, characteristics of the women were similar, with a higher percentage of women in the three urban hospitals covered by insurance schemes, and 88% of the women at the rural county hospital paying out of pocket fees [1]. Caesarean section rate was high and ranged from 70% at the district hospital through to 28% at the county hospital; and the pattern was similar in 1999 (table 3).

#### Evidence-informed practice indicates "avoid as routine"

In 1999, practices that were routine (defined as more than 65% of vaginal births) during normal delivery and contrary to current evidence and international standards included episiotomy (all four hospitals); pubic shaving, rectal examination to monitor labour and electronic fetal heart monitoring (3 hospitals); and enema on admission was common in the district hospital. In 2003 no changes were apparent for episiotomy at the specialist, district or county hospitals, but this procedure increased significantly at the city MCH hospital; pubic shaving declined from 100% to 45% at the city MCH hospital; rectal examination for monitoring labour was stopped at the specialist hospital; and enema use declined significantly from 54% to 3% in the district hospital where this was previously common practice. During the period, electronic fetal monitoring remained high at the urban hospitals, and, in the rural county hospital, increased from 1% to 27% (see table 4).

#### Evidence-informed practice indicates "encourage as routine"

In 1999, continuous support during labour was uncommon in the specialist and county hospitals and mobility during labour uncommon at the county hospital; for the district hospital, continuous support was widespread and mobility allowed in about half of the women. In 2003, continuous support showed little change in all facilities; mobility increased significantly at the specialist and city MCH hospitals, but declined significantly at the district hospital. Most births were in the supine position for all facilities, and this did not change.

### Provider opinion of influences on their practice over four years

We identified three main influences on provider practice from the semi-structured interviews with the providers.

#### Hospital directors

The hospital director communicating international practice standards to doctors and midwives was important in reducing pubic shaving at the city MCH hospital, where there is frequent collaboration and exchange with US hospitals and experts. The director explained,

*"over the last three years we have many changes, every year, twice a year we invite experts from abroad to come to the hospital and we send many doctors abroad. The mutual exchange process opens the mind and this opinion is similar to what we were told three years ago – reducing interventions and promoting natural delivery"*.

Doctors and midwives mentioned that this contact had brought 'new opinions' and helped them to think about changing practice, as one midwife described,

"the director told us that they [US hospital] don't advocate pubic shaving, enemas and so on, US experts bring us many new opinions. We should begin to adapt. Our director said we can try – if we don't shave, what will happen?"

The midwife in charge explained that during the period of this study (1999–2003) the director of obstetrics had instigated a randomised controlled trial (in 2002) of women entering the labour room to determine the effects of shaving. She mentioned that the infection rate appeared to be no different among those who had been shaved and those who had not, so they had prepared to stop shaving all women.

At the specialist hospital, the hospital director's efforts to disseminate international practice standards appeared to be influential in changing the hospital policy for rectal examination; providers' comments suggested that advocacy by hospital 'leaders' had initiated change in the whole obstetric department. One doctor explained,

*"when I came to labour ward I was told I should do vaginal exam instead of rectal exam because now the antibiotics have good effect and they [doctors in the ward] feel that the vaginal examination is more accurate. The director told me these things, and I also feel like that. We use sterile gloves to perform vaginal examination and we have no problems"*.

The director of obstetrics commented,

*"the change was in fact due to the change of service providers' attitudes. In hospitals abroad, service providers all use vaginal examination. In our experience we thought vaginal examination was liable to infection; in fact rectal exam is dirty and often not accurate for assessing dilation, also women do not feel very comfortable. Otherwise when the woman is in labour the vagina is loose so they are more likely to accept vaginal exam. We found after the sterilization [staff awareness and use of sterile gloves] there was no higher infection rate so we changed it"*.

At the district hospital, the director's opinion seemed to be an important influence on the continued use of rectal examination:

*"rectal exam has been used for a long time and has become tradition. We also want to change it, but the vaginal exam requires strict sterilization [use of clean methods]. If it is not strict, then with more vaginal exams, more women will have fever"*.

She explained how she wanted to conduct a trial in her hospital to determine the effect, and suggested that,

*"if the result is similar for the two examinations then I can make changes, otherwise I don't want to change"*.

#### Medical regulation and municipal routines

Providers frequently mentioned the recent enforcement of medical regulations as a contributing factor to changed practice. For example, episiotomy practice: at the city MCH hospital, use of episiotomy increased significantly between 1999–2003, despite implementation of a policy to 'protect the perineum'. Providers at this hospital explained that a restrictive policy was hard to implement as the 'Medical Accidents Punishing Regulations', introduced in 2002, categorise third degree perineal trauma as a medical accident. A doctor at the county hospital made similar comments:

*"the trend [for episiotomy] is increasing because of the fear of third degree tears, which run the risk of medical conflict, and under such pressure they will widen the implications for episiotomy"*.

Other doctors explained in more detail their fear of causing a 'medical accident', for example:

*"I think all primigravidae should have episiotomy. If you are sure the baby is small then episiotomy is not necessary, but if I don't do an episiotomy and the woman tears, then it is my mistake"*.

Providers often mentioned the need to follow municipal routines at hospital level, as well as awareness of medical regulation, as an important influence on practice. The director of obstetrics at the district hospital mentioned that following municipal routine was a key reason for reducing the use of enemas:

*"now the policy is to follow the municipal routine because of the risk of being sued"*.

A midwife at the county hospital described how she thought following municipal routines could help realise practice change across whole departments:

*"if only one person changes it will not have a big effect but if you do not follow the routine for a patient, then if something happens, it will be your problem. If all doctors' opinion can change, then this situation will not happen"*.

#### Women's preferences

Staff at the district hospital stated that women's preference was now an important consideration in enema use, as one midwife explained,

*"if pregnant women don't want an enema, then we don't give them an enema, but before we only didn't give enemas when there was a contra- indication"*.

Another doctor suggested giving women more decision-making power changed the way they practice:

*"now we have a new opinion – to give patients the right to make decisions themselves, and if we want to do something for her we should give an explanation"*.

Some doctors at the urban hospitals mentioned that women's choice was an important influence on the number of Caesarean deliveries performed, and they thought this was due to the fact that some women insist on caesarean section, despite their efforts to persuade them to deliver normally. The director at the specialist hospital believed that as a doctor she should advocate for normal delivery, but

"if the woman insists on CS, the doctor should respect the woman's choice, but clearly explain the risks of the operation to the woman and let her sign an informed consent form".

The doctor in charge at the city MCH hospital thought that for women who insist on CS it is difficult to change their mind:

"for some women a psychological problem exists. If she thinks that she can't accept vaginal delivery absolutely, she will feel uncomfortable in her mind".

## Discussion

This is a small descriptive study including two time periods at four sites only; while it is likely that obstetric practice is similar in other provinces due to the standardised national guidelines and medical regulations, the findings and implications relating to factors influencing practice changes cannot necessarily be generalised to hospitals in other provinces or nationwide. We selected urban centres with a good reputation as these centres are expected to provide optimal performance in the Shanghai region.

A notable finding is the high Caesarean section rate across the four study sites; a pattern that showed little change from 1999–2003. There is an indication that self-decision making for Caesarean section increased over the four years at the study hospitals [30], and there is limited evidence from elsewhere in China that demand for Caesarean section is associated with women's belief that Caesarean delivery is safe for newborns and is less painful for the woman [31]. The extent to which maternal requests for caesarean delivery without medical indication are responsible for the increasing rate of caesarean sections is widely debated internationally [32-34]; a Cochrane review is planned to inform this debate by comparing the effects of planned vaginal versus planned caesarean delivery [35].

We included in the analysis of obstetric procedures only women who delivered vaginally. It is possible that some of the procedures we measured may have influenced whether women delivered vaginally or not; however, the objective of our study was simply to explore routine procedures in vaginal births.

The study demonstrates practice changed in a few obstetric procedures over four years. There appeared to be no pattern in what procedure changed and which hospital it changed in. The direction of the change was, in all but one procedure, towards more evidence-based practice, with uncomfortable procedures such as enemas or rectal examinations being abandoned.

The changes at each hospital were different and the process of change was complex. We initially thought that the feedback to the each hospital after the 1999 survey was responsible for change, but the qualitative data demonstrated that there were multiple factors and the survey appeared not to play a part although it could have done indirectly by influencing the hospital director. Whilst it is to be expected that the hospital director is particularly influential in clinical practice, their influence in these hospitals appears high, and indicates that their endorsement of evidence-based change, and their interpretation of the evidence around each single procedure, is important. The change of hospital directors during the study period at the specialist and county hospitals might have played a particularly important role in the significant practice changes observed; at the specialist hospital, qualitative data suggest that the director's efforts to disseminate international practice standards was influential in changing policy for rectal examinations. It seems that the uptake and enthusiasm for change comes from the directors and other research on 'opinion leaders' has demonstrated this [14,36].

It is interesting that legislation to protect the women has actually led obstetricians to avoid reducing routine episiotomies. The legislation made perineal tear a clinical error and episiotomy rates stayed high; the qualitative data indicated that this was in spite of the evidence that routine episiotomy does not prevent third degree tears [23]. This change in practice is the equivalent to defensive practice-to avoid litigation rather than in the best interests of the patient.

There appeared to be some emerging evidence from the interviews of a more informed client base questioning practice; on the other hand, obstetric staff, exposed to evidence that does not support routine enemas may actually start offering choice. It is clear from work in South Africa that midwives informed about evidence based standards are frequently keen to abandon an enema, because they make a mess and create work [37]. It should also be considered that providers are obliged to give women more choice during childbirth, as this is what hospital policy dictates [20,38].

None of the interviews identified the dissemination of evidence-based concepts or information as central to the changes that took place. We cannot therefore easily determine any effect of the regional dissemination activities outlined in table 1. However, this does not rule out that they are important in sensitising clinicians to the need for change; and the mention in the qualitative data of the use of randomised trials to evaluate practice at the city MCH and district hospitals is an important indication of evidence-informed thinking. Similar findings were reported in the Leeds University maternity audit project, which measured compliance with evidence-based recommendations in 20 UK maternity units. The study found a shift in practice in line with evidence over an eight year period (1988 -1996), with little evidence of any planned dissemination or implementation activities at the maternity units; the authors comment on the difficulty in attributing the change to availability of evidence through the Cochrane Collaboration and national dissemination activities [3].

## Conclusion

The study shows that there has been change in some obstetric practices in these selected hospitals. The strongest influences appear to be the hospital directors, and they were influenced by experts from abroad and practice in other countries, towards evidence-informed practice; and medical legislation in the country, which happened on this occasion to encourage a move away from evidence-based practice. Change does not appear to be directly related to the promotion of evidence-based practice in the region, although it may contribute indirectly.

## Competing interests

The author(s) declare that they have no competing interests.

## Authors' contributions

XQ designed the study, managed the research process and drafted the paper. HL collected and analysed data as part of a Masters thesis and helped write the paper. HS contributed to the objectives, design, data analysis, interpretation and writing. JL conducted the statistical analysis. PG contributed to the concept, design, provided methodological support and helped write the paper.

## Pre-publication history

The pre-publication history for this paper can be accessed here:



## Figures and Tables

**Table 1:** National and local Shanghai initiatives to promote evidence-based obstetric care from 1999

1. Women's Peace Hospital launched the Chinese version of the WHO Reproductive Health Library containing systematic reviews and commentaries.
2. The World Health Organization (WHO) and UNICEF promotion of mother- friendly initiatives, to support a wellness model of maternity care to improve birth outcomes and substantially reduce costs which had reliable evidence as a base [39].
3. The women's health care division of China Preventive Medicine Association (CPMA):
▪ emphasised humane care and the reduction of interventions during childbirth through their national academic conference in Guangzhou in 2002 [40]
▪ developed the Advanced Learning Support in Obstetrics (ALSO) training program in China to help obstetricians grasp the latest knowledge and skills; the ALSO textbook has been translated into Chinese [41]
▪ initiated a project in 13 maternal and child health hospitals in 6 cities to support normal birth and promote evidence-based obstetric care that is humane and women-friendly [42].
4. Evidence from systematic reviews in reproductive health were summarized and translated, to recommend change to service providers, by researchers at Fudan University School of Public Health [43].

**Table 2:** Characteristics of participants by study site in 1999 and 2003

	Specialist		City MCH		District		County	
	1999	2003	1999	2003	1999	2003	1999	2003
N	150	162	150	160	150	228	149	161
Mean age ± SD	28.2 ± 4.6	27.9 ± 3.9	27.7 ± 3.6	27.9 ± 3.9	28.5 ± 4.9	27.9 ± 4.7	24.5 ± 2.7	24.3 ± 3.5
High school education^† ^(%)	119 (79)	126 (78)	130 (87)	151 (94)	116 (77)	164 (72)	40 (27)	48 (30)
First delivery (%)	132 (88)	147 (91)	143 (95)	155 (97)	139 (93)	196 (86)	139 (93)	145 (90)
Self payment (%)*	59 (39)	71 (44)	39 (26)	46 (29)	77 (51)	129 (57)	122 (82)	142 (88)

^† ^Women who had completed education up to age 18.

*Payment of hospital fees out of pocket rather than via government, labour or medical insurance schemes

**Table 3:** Pregnancy outcome in 1999 and 2003 by hospital in women surveyed

	Specialist		City MCH		District		County	
	1999	2003	1999	2003	1999	2003	1999	2003
**Pregnancy outcome**								
N	150	162	150	160	150	228	149	161
Spontaneous vaginal (%)	60 (40)	66 (41)	71 (47)	88 (55)	41 (27)	68 (30)	99 (66)	110 (68)
Vacuum/forceps (%)	15 (10)	11 (7)	11 (7)	7 (4)	0 (0)	0 (0)	6 (4)	6 (4)
Elective CS (%)	41 (27)	68 (42)	36 (24)	45 (28)	87 (58)	135 (59)	28 (19)	24 (15)
Emergency CS (%)	34 (23)	17 (11)	32 (21)	20 (13)	22 (15)	25 (11)	16 (11)	21 (13)

**Table 4:** Obstetric practices during vaginal delivery (including vacuum and forceps delivery) in 1999 and 2003 by hospital in women surveyed

	Specialist		City MCH		District		County	
Vaginal deliveries (women)	1999	2003	1999	2003	1999	2003	1999	2003
	n = 75	n = 77	N = 82	n = 95	n = 41	n = 68	n = 105	n = 116
**Avoid as routine†**								
Episiotomy (%)	70 (93)	74 (96)	74 (90)	94 (99)*	36 (88)	63 (93)	68 (65)	88 (76)
Pubic shaving	0 (0)	0 (0)	82 (100)	43 (45)**	39 (95)	68 (100)	98 (93)	102 (88)
Rectal examination	71 (95)	0 (0)**	0 (0)	0 (0)	39 (95)	68 (100)	103 (98)	105 (91)*
Electronic FHR	75 (100)	75 (97)	76 (93)	87 (92)	37 (90)	65 (96)	1 (1)	31 (27)**
Enemas	1 (1)	0 (0)	1 (1)	0 (0)	22 (54)	2 (3)**	0 (0)	5 (4)
**Encourage as routine†**								
Companionship during labour^1^	6 (8)	12 (16)	30 (37)	45 (49)	38 (93)	62 (93)	8 (17)	9 (9)
Mobility during labour^1^	27 (37)	60 (78)**	19 (23)	47 (51)**	23 (56)	19 (28)**	8 (17)	31 (29)
Non-supine position in Labour	0(0)	1(1)	0(0)	1(1)	0(0)	0(0)	3(3)	1(0.9)

**† **Procedures categorized with reference to research evidence from Cochrane Systematic Reviews

^1 ^Some women were directly sent to delivery room, so for companionship and mobility the sample size at the City MCH, District and County hospital in the follow-up study is 92, 67 and 106 respectively in 2003. In the 1999 study, the sample size of the Specialist and the County hospitals was 74 and 46 respectively.

* P-value < 0.05 (Chi^2 ^test)

** P-value < 0.01 (Chi^2 ^test)

Data source: exit interviews with postpartum women

**Table 5:** What this study adds

**What is already known on this subject**
A gap between evidence and practice in managing normal labour is common in many countries.
Practice change is often influenced by opinion leaders.
**What this study adds**
In some hospitals in Shanghai there is a substantive gap between best practice derived from reliable evidence and actual practice.
Over four years (1999–2003), obstetric practice in these hospitals became more evidence-informed, with substantive changes in some routine procedures.
Change towards evidence-informed practice was mainly influenced by the hospital director; change away from evidence-based practice was the result of legislation about medical accidents.
Regional seminars about best practice seemed to have had little direct influence on change.

## References

[B1] Qian X, Smith H, Zhou L, Liang J, Garner P (2000). Evidence based obstetrics in four hospitals in China: An observational study to explore clinical practice, women's preferences and provider's views. BMC Pregnancy and Childbirth.

[B2] Smith H, Brown H, Hofmeyr GJ, Garner P (2004). Evidence-based obstetric care in South Africa – influencing practice through the 'Better Births Initiative'. S Afr Med J.

[B3] Wilson B, Thornton JG, Hewison J, Lilford RJ, Watt I, Braunholtz D, Robinson M (2002). The Leeds University maternity audit project. Int J Qual Health Care.

[B4] Kozak LJ, Weeks JD (2002). US trends in obstetric procedures 1990–2000. Birth.

[B5] Althabe F, Belizán JM, Bergel E (2002). Episiotomy rates in primiparous women in Latin America: hospital based descriptive study. Br Med J.

[B6] Madume-Butshe A, Dyall A, Garner P (1998). Routine episiotomy in developing countries. Br Med J.

[B7] Cai WW, Marks JS, Chen CH, Zhuang YX, Morris L, Harris JR (1998). Increased caesarean section rates and emerging patterns of health insurance in Shanghai, China. Am J Public Health.

[B8] Savage W (2000). The Caesarean section epidemic. J Obstet Gynaecol.

[B9] Leung GM, Lam TH, Thach TQ, Wan S, Ho LM (2001). Rates of Cesarean births in Hong Kong: 1987–1999. Birth.

[B10] Comparative analysis of status of child health care in Beijing, Tianjin, Shanghai and Chongqing. http://www.few.gov.cn/jcbg/kygg8.htm.

[B11] Jiazeng H (1998). The trend of service delivery model in the world. Maternal and Child Health Care of China.

[B12] NHS Centre for Reviews and Dissemination (1999). Getting evidence into practice. Effective Health Care Bulletin.

[B13] Grol R (1997). Beliefs and evidence in changing clinical practice. Br Med J.

[B14] Lomas J, Enkin M, Anderson G, Hannah W, Vayda E, Singer J (1991). Opinion leaders vs audit and feedback to implement practice guidelines. JAMA.

[B15] Grol R (2001). Improving the quality of medical care: building bridges among professional pride, payer profit, and patient satisfaction. JAMA.

[B16] Eccles M, Grimshaw J, Walker A, Johnston M, Pitts N (2005). Changing behaviour of healthcare professionals: the use of theory in promoting the uptake of research findings. J Clin Epidemiol.

[B17] Plsek P, Greenhalgh T (2001). The challenge of complexity in health care. Br Med J.

[B18] Greenhalgh T, Robert G, Macfarlane F, Bate P, Kyriakidou O (2004). Diffusion of innovations in service organisations: systematic review and recommendations. Milbank Q.

[B19] Eisenberg J (2002). Globalise the evidence, localise the decision: evidence- based medicine and international diversity. Health Aff (Millwood).

[B20] Medical Accidental Punishing Regulations, 2002. http://news.xinhuanet.com/zhengfu/2002-04/15/content_358058.htm.

[B21] Basevi V, Lavender T (2000). Routine perineal shaving on admission in labour. Cochrane Database Syst Rev.

[B22] Cuervo LG, Rodríguez MN, Delgado MB (1999). Enemas during labour. Cochrane Database Syst Rev.

[B23] Carroli G, Belizan J (1999). Episiotomy for vaginal birth. Cochrane Database Syst Rev.

[B24] Hodnett ED, Gates S, Hofmeyr GJ, Sakala C (2003). Continuous support for women during childbirth. Cochrane Database Syst Rev.

[B25] Gupta JK, Hofmeyr GJ (2004). Position for women during second stage of labour. Cochrane Database Syst Rev.

[B26] Lewis L, Webster J, Carter A, McVeigh C, Devenish-Meares P (2002). Maternal positions and mobility during first stage labour (protocol). Cochrane Database Syst Rev.

[B27] Dean AG, Dean JA, Columbier D, Brendel KA, Smith DC, Burton AH (1995). Epi Info, Version 6: a word processing, database and statistics programme for public health on IBM-compatible microcomputers.

[B28] (2001). SPSS for Windows, Rel. 11.5.

[B29] Ritchie J, Spencer L, Bryman A, Burgess RB (1994). Qualitative data analysis for applied policy research. Analysing qualitative data.

[B30] Liang H, Qian X, Liang J (2004). A study on evidence-based obstetric care among four types of hospitals. Masters thesis.

[B31] Sun CY, Wang YZ (2000). The analysis of the reason for high Cesarean section rate. Journal of Harbin Medical University.

[B32] Minkoff H, Chervenak F (2003). Elective primary cesarean delivery. N Engl J Med.

[B33] Penna L, Arulkumaran S (2003). Cesarean section for non-medical reasons. Int J Gynaecol Obstet.

[B34] Wagner M (2000). Choosing cesarean section. Lancet.

[B35] Lavender T, Hofmeyr GJ, Neilson JP, Kingdon C, Gyte G (2004). Caesarean section for non-medical reasons at term (Protocol). Cochrane Database Syst Rev.

[B36] Thomson O'Brien MA, Oxman AD, Haynes RB, Davis DA, Freemantle N, Harvey EL (1999). Local opinion leaders: effects on professional practice and health care outcomes. Cochrane Database Syst Rev.

[B37] Smith HJ (2002). Implementing evidence based obstetrics in a middle income setting: a qualitative study of the change process. PhD thesis.

[B38] The guidelines of medical record writing (probation). http://www.sunhaipeichang.com/Article/shpch/ylshg/ylfl/ylgchfl/200501/6529.html.

[B39] The Baby Friendly Hospital Initiative (UNICEF). http://www.unicef.org/programme/breastfeeding/baby.htm.

[B40] National Working Committee on Children and Women (Humane service for delivery is most natural). http://www.people.com.cn/GB/kejiao/42/152/20020614/752443.html.

[B41] American Academy of Family Physicians (2002). Advanced Learning Support in Obstetrics.

[B42] Shen R, Huang X, Xiang X (2002). Protection, promote and support normal birth. Maternal and Child Health Care of China.

[B43] Research progress in effective health care – summary of abstracts of systematic reviews. http://www.shmu.edu.cn/effect/index.html.

